# Quasi-reference electrodes in confined electrochemical cells can result in *in situ* production of metallic nanoparticles

**DOI:** 10.1038/s41598-018-20412-2

**Published:** 2018-01-31

**Authors:** Rukshan T. Perera, Jacob K. Rosenstein

**Affiliations:** School of Engineering, Brown University, 184 Hope Street, Providence, RI 02912 USA

## Abstract

Nanoscale working electrodes and miniaturized electroanalytical devices are valuable platforms to probe molecular phenomena and perform chemical analyses. However, the inherent close distance of metallic electrodes integrated into a small volume of electrolyte can complicate classical electroanalytical techniques. In this study, we use a scanning nanopipette contact probe as a model miniaturized electrochemical cell to demonstrate measurable side effects of the reaction occurring at a quasi-reference electrode. We provide evidence for *in situ* generation of nanoparticles in the absence of any electroactive species and we critically analyze the origin, nucleation, dissolution and dynamic behavior of these nanoparticles as they appear at the working electrode. It is crucial to recognize the implications of using quasi-reference electrodes in confined electrochemical cells, in order to accurately interpret the results of nanoscale electrochemical experiments.

## Introduction

New insights into nanoscale chemical systems have come hand in hand with experimental techniques that can probe ever-smaller volumes, but traditional physiochemical models cannot necessarily be applied to systems with nanoscale dimensions. This fact, combined with the availability of new fabrication methods to prepare nanoscale electrodes and nanoconfined electrochemical devices, has inspired a new branch of study referred to as “nanoelectrochemistry”^1–6^.

Physical size reductions yield important benefits such as reduced background noise, reduced *iR* drop, and fast mass transport rates, and these features can be used to study phenomena at length scales approaching the dimensions of single molecules^7–15^. In addition to fundamental studies of nanoparticle electrocatalysis^16–18^, single nanoparticle detection^19–22^, single-molecule sensing^23–26^, and electrochemical imaging^27–30^, miniaturized electrochemical systems have found applications in energy conversion and storage^31–33^.

However, the extreme sensitivity required by nanoscale electrochemical measurements can also open the door to new complications. Numerous experimental hazards must be carefully managed in order to acquire meaningful data sets which are large enough to have sufficient statistical power. These include challenges with substrate cleanliness, background noise, sample purity, and nanoelectrode fouling, among others. It is important to understand the mechanisms behind these complications, in order to be in a better position to avoid them.

In an ideal three-electrode electrochemical cell, the reference electrode is isolated from the bulk solution using a glass frit or salt bridge, and the counter electrode is positioned far from the working electrode. However, for reasons of size, cost, and complexity, miniaturized analytical devices often do not have this luxury. Instead, many systems use a non-isolated “quasi-reference” electrode, such as a simple Ag/AgCl wire. In some cases it is even common to eliminate the third electrode, and to instead use a simpler 2-electrode cell which balances the working electrode with a single “quasi-reference counter electrode” (QRCE). Such arrangements can be justified if the current measured is very small and the surface area of the QRCE is relatively large compared to the WE.

However, as the distance between the WE and QRCE decreases, or the duration of an experiment increases, the diffusion of redox species generated at the QRCE to the working electrode may not be negligible. For example, a recent article reported artifacts observed in a lab-on-a-chip microelectrode arrays when non isolated Ag/AgCl reference electrodes are used^34^. It would be valuable to understand the origin of such artifacts, and the physical processes that guide them.

In this study, we use a scanning nanopipette contact^35–40^ to show that a QRCE in a miniaturized electrochemical cell can lead to *in situ* nanoparticle generation even in the absence of any precursor. We explore the key experimental factors responsible for this phenomenon. This is a critical practical observation that should not be ignored, as it will ultimately determine the achievable detection limits in nanoelectrochemical experiments.

## Results and Discussion

**Figure 1:**
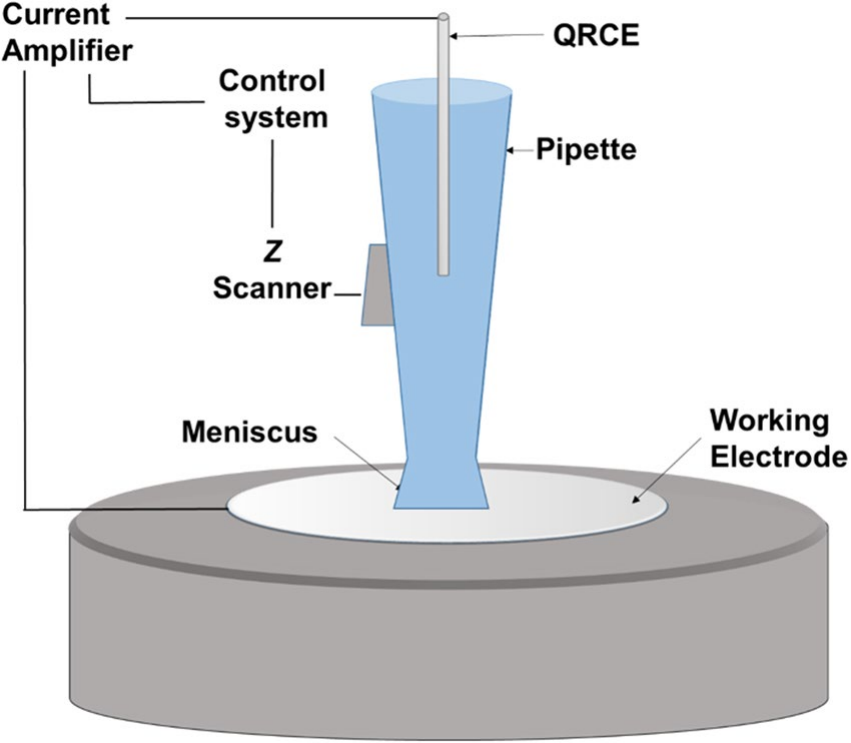
Schematic representation of the experiment (not to scale). A glass capillary is carefully positioned so that its tip forms a nanometer-scale liquid meniscus with a working electrode.

All experiments were performed using a scanning nanopipette contact setup (Fig. 1). Briefly, a quartz capillary with ~40 nm diameter is fabricated with laser-assisted pipette puller. The pipette is filled with electrolyte and held by a nanopositioning stage directly above a working electrode. A QRCE is inserted into the pipette, and a bias voltage (−0.2 V) is applied between the QRCE and the working electrode. The pipette is lowered in 100-nm increments until its tip forms a liquid meniscus contact with the working electrode. This meniscus is detected by a characteristic current spike observed during the contact due to electric double layer charging at the interface. Once a meniscus is established, the pipette movement is halted. All potentials are reported as V vs QRCE used in the specific experiment.

**Figure 2:**
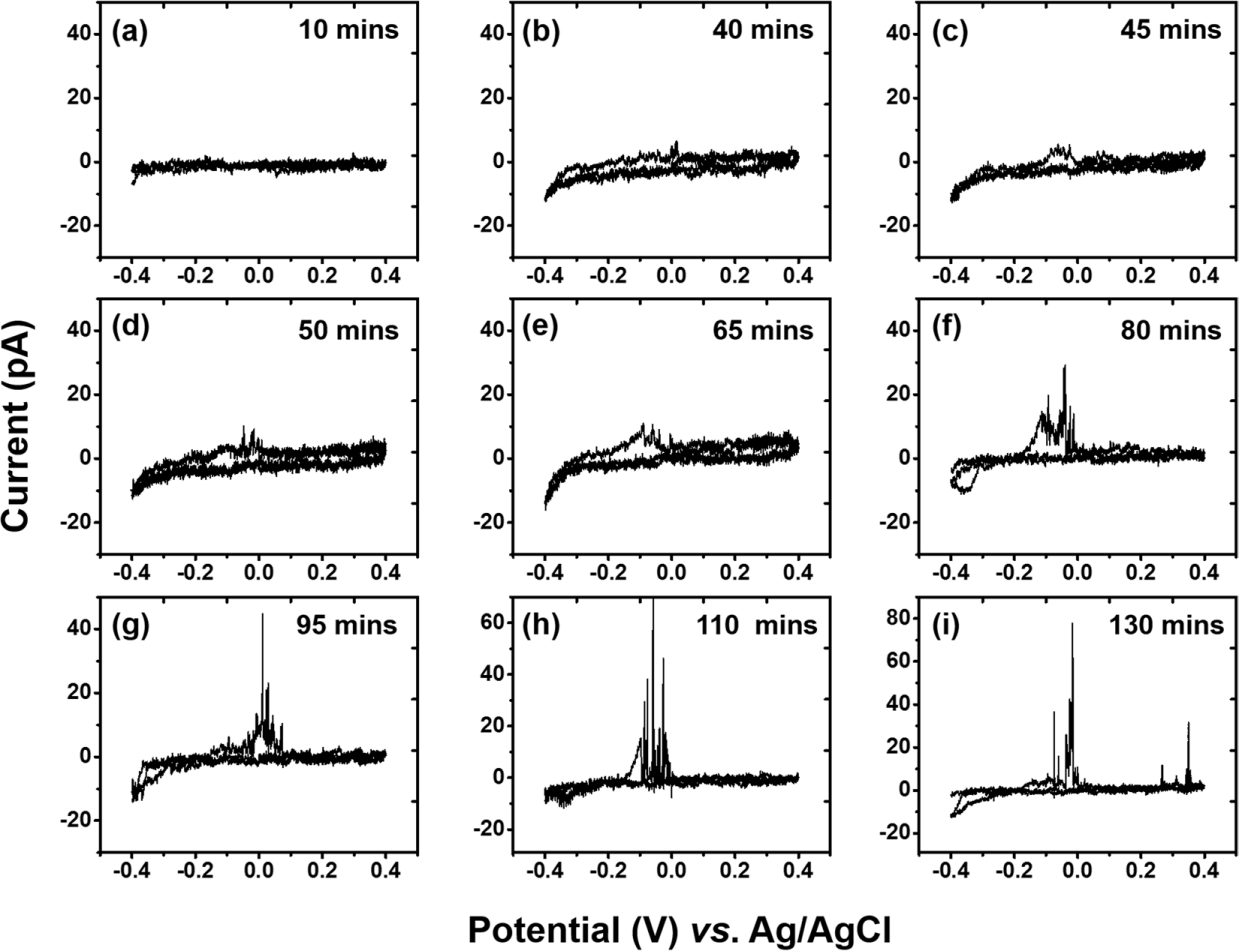
Cyclic voltammograms showing progress of deposition-dissolution behavior over the time. Representative CVs are shown after (**a**) 10 min (**b**) 40 min (**c**) 45 min (**d**) 50 min (**e**) 65 min (**f**) 80 min (**g**) 95 min (**h**)110 min (**i**) 130 min after initial contact on GC electrode vs Ag/AgCl QRCE. All voltammograms were recorded at 0.1 V/s.

First, we utilized cyclic voltammetry to evaluate the interference of Ag/AgCl QRCE electrodes commonly used for scanning pipette measurements. No electroactive species were present in the electrochemical cell, and we selected 0.1 M LiCl as representative of chloride electrolytes commonly used with an Ag/AgCl QRCE (to obtain a stable potential by maintaining the reaction: AgCl_(s) _+ e $\leftrightarrows$ Ag_(s)_ + Cl^−^_(aq)_). A glassy carbon (GC) electrode was used as the working electrode. Once a meniscus contact was established, cyclic voltammograms were performed continuously at 0.1 V/s for Ag/AgCl QRCE for a period of 130 min, as shown in Fig. 2. Initially, the *i-V* curve did not show any significant oxidation or reduction current. However, after 40 min of continuous measurements (Fig. 2b), signs of redox activity were observed. This redox behavior increased in prominence until 110 min had elapsed, with an oxidation peak near −0.05 V and crossover point ~0.2 V. The shapes of these cyclic voltammograms are reminiscent of metal deposition and stripping^41^, and sharp current transients just above after bulk oxidation potential suggest the presence of discrete oxidizable nanoparticles. These transients will be explored in more detail in later sections.

**Figure 3:**
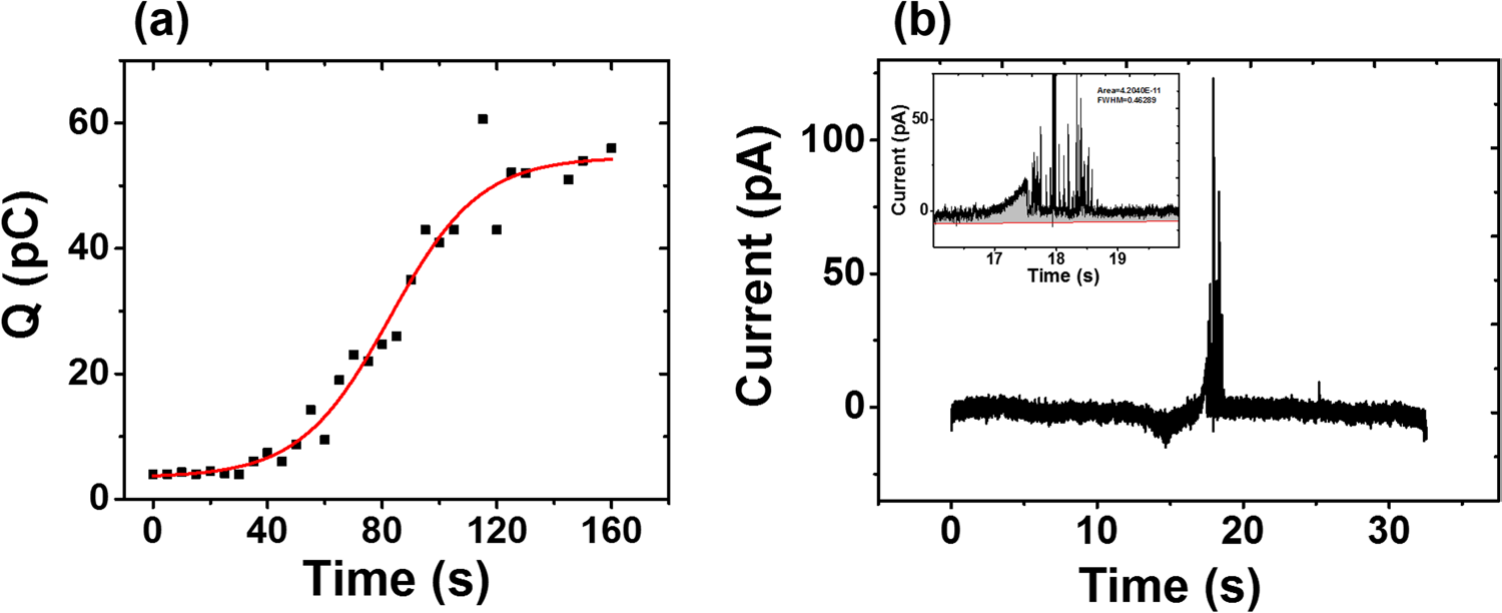
Total oxidation charge for a single voltammetry scan, calculated over the course of 160 min for Ag/AgCl QRCE and a GC working electrode. (**a**) Integrated oxidation charge versus time elapsed. The data is fit to a Hill-1 sigmoidal model. (**b**) An example *i-t* trace from a voltammogram after 110 min. Inset: expanded sections of the oxidation current peaks, showing the integrated charge area. Additional *i-t* traces used to plot (**a**) are shown in Fig. S2.

We analyzed the total oxidation charge transferred during a voltammetry sweep, which exhibits a sigmoidal curve as time elapses (Fig. 3). Here we performed experiments for 160 min, and similarly to Fig. 2, no electrochemical oxidation is visible until after approximately 40 min. The calculated oxidation charge in each scan increases steadily over the time until settling at ~60 pC (~0.6 femto mols) after 130 min. We hypothesize that this asymptotic curve is due to a reduced nucleation rate caused by depletion of metal ions in the vicinity of the working electrode, leading to diffusion-limited conditions for forming nanoparticles from dissolved ions.

### Stripping behavior depends on the QRCE

**Figure 4:**
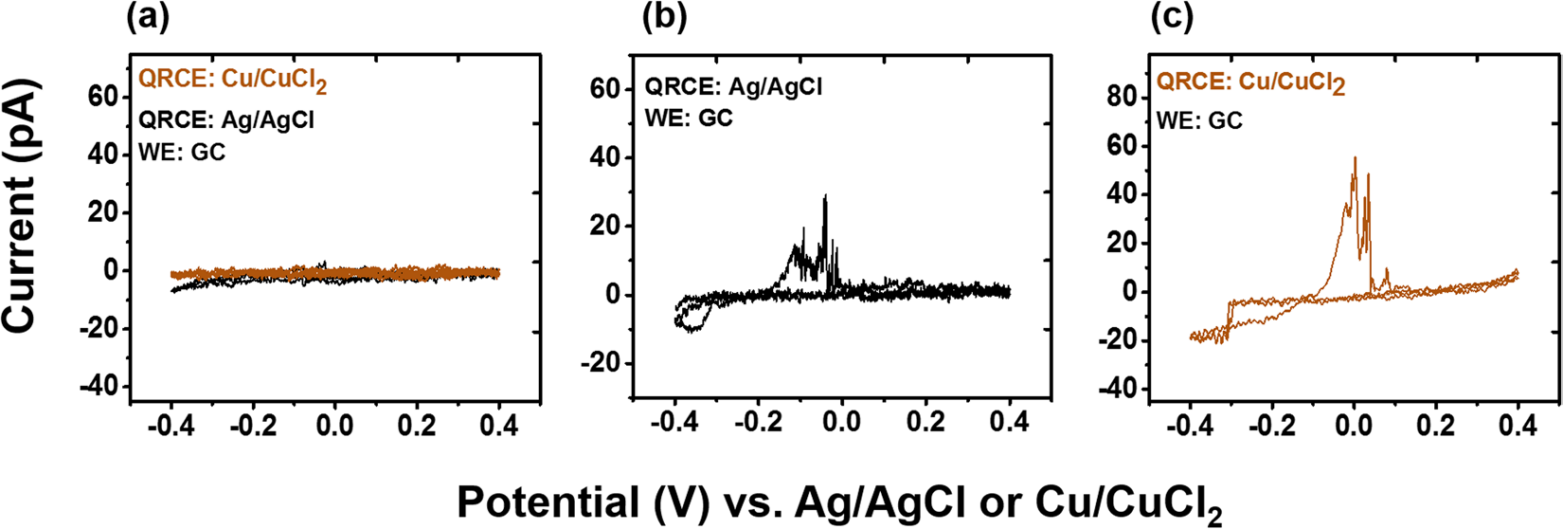
Cyclic voltammograms recorded with a GC working electrode after ~110 min vs Ag/AgCl and Cu/CuCl_2_ are indicative of deposition and dissolution. CVs are recorded (**a**) after initial contact, (**b**) vs Ag/AgCl after ~110 min, and (**c**) vs Cu/CuCl_2_ after ~110 min. All voltammograms were recorded at 0.1 V/s.

To test how this stripping behavior at the working electrode may change with other QRCEs, we used another commonly used Cu/CuCl_2_ QRCE with the same experimental set up under similar experimental conditions. The observed results are compared with Ag/AgCl in Fig. 4. As indicated in Fig. 4(a), neither QRCE shows visible oxidation or reduction immediately after contact. However, as the time progressed, similar deposition-stripping behavior was observed with the Cu/CuCl_2_ QRCE, as shown in Fig. 4(c).

### Proposed mechanism for the *in situ* generation of nanoparticles

It is worthwhile to emphasize once again that the electrolyte used in these experiments did not contain any electroactive species or added metal ions. The redox activity is also not due to dissolved gas and is instead characteristic of metal ion dissolution and deposition. Therefore, we hypothesize that silver and copper ions are released from the QRCE, and are responsible for the observed redox activity. The reversible reactions at the QRCE are as follows,

$${{\rm{AgCl}}}_{({\rm{s}})}+{\rm{e}}\leftrightarrows {{\rm{Ag}}}_{({\rm{s}})}{{+\mathrm{Cl}}^{-}}_{({\rm{aq}})}$$

$${{\rm{C}}{\rm{u}}{\rm{C}}{\rm{l}}}_{2({\rm{s}})}+2{\rm{e}}\leftrightarrows {{\rm{C}}{\rm{u}}}_{({\rm{s}})}{{+2{\rm{C}}{\rm{l}}}^{-}}_{({\rm{a}}{\rm{q}})}$$

**Figure 5:**
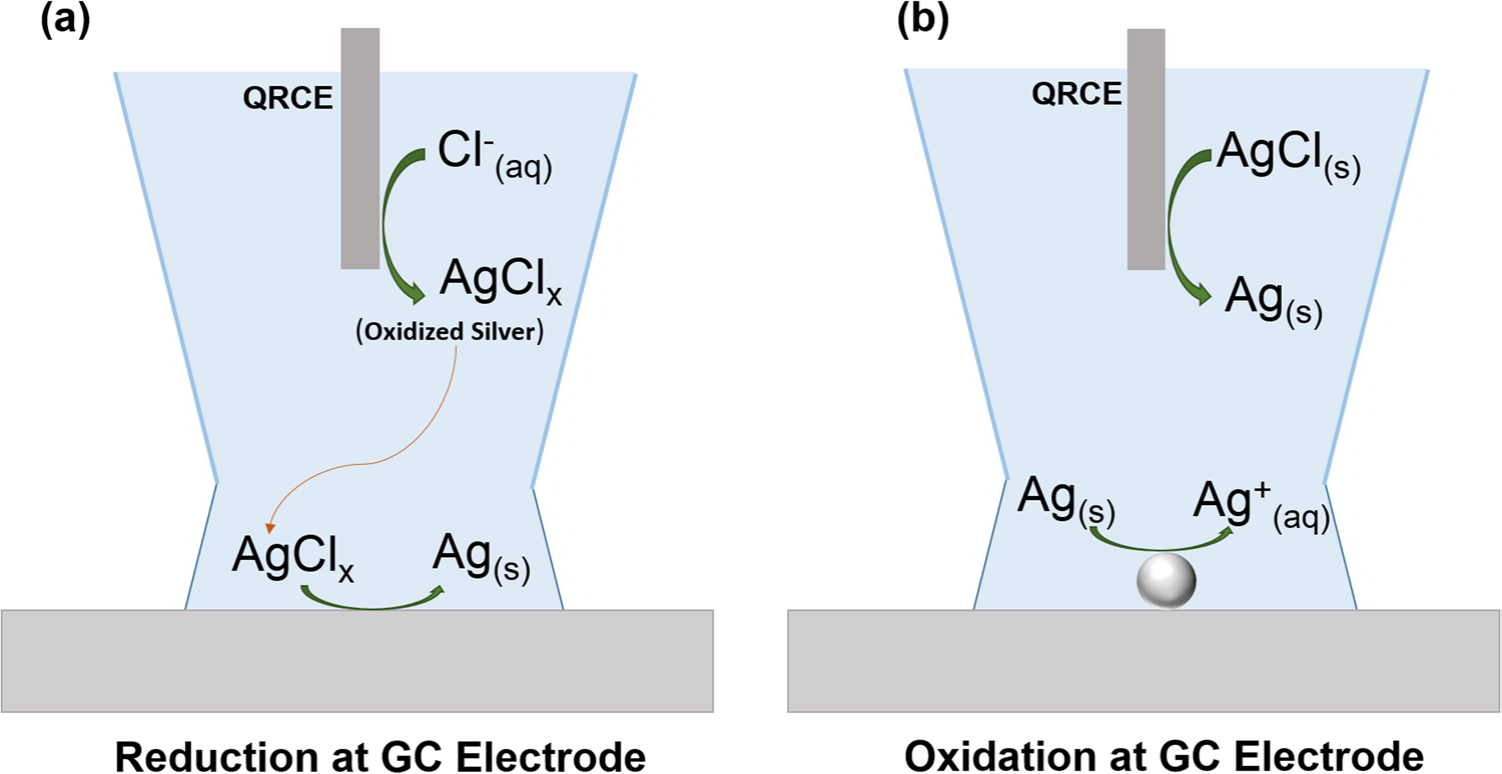
Schematic representation of a proposed mechanism for *in situ* particle generation and oxidation. (**a**) Reduction potentials at the GC electrode trigger oxidized Ag (AgCl_x_ where x can be 1–4) formation at the Ag/AgCl QRCE. The oxidized Ag diffuses to the GC electrode, where it undergoes reduction to form Ag nanoparticles (Ag NPs). (**b**) At oxidation potentials, the Ag NPs are oxidized.

At negative applied potentials, reduction current at the working electrode is balanced by an oxidation reaction at the QRCE. At sufficient reduction potentials vs QRCE, oxidized Ag/Cu formation is favorable. The fate of resulting Ag^+^ ions can vary depending on experimental conditions. For example, in the presence of chloride ions, Ag^+^ can complex with multiple chloride ions^42,43^. Depending on the strength of the adsorption to the QRCE and the solubility of the complexes formed, these oxidized metal ions can diffuse into the bulk electrolyte solvent as illustrated in Fig. 5. These processes lead to instability of the reference electrode and eventually cause its potential to drift. In a larger-scale experiment, small amounts of QRCE instability may be tolerable. However, in miniaturized experimental cells, metal ions generated at the QRCE may eventually reach the working electrode and be reduced into metal NPs. The electrolyte volume in our demonstration is ~30 µL, and can vary between ~30–160 µL for comparable capillary probe based electrochemical setups. The distance between the QRCE and the working electrode for glass capillary systems is typically between 1–5 cm, although for some microfluidic devices or for screen printed electrodes, this distance can be much smaller. Consumption of metal ions at the working electrode produces a chemical gradient which further draws ions towards the electrode, resulting more and more deposition at the working electrode during voltage cycling. We observe NP generation at the working electrode sooner than would be expected from simple diffusive transport, and it is reasonable to expect that the mass transport of metal ions may be enhanced by nonlinear diffusion within the tapered nanopipette geometry^44^, natural convection from density gradients^45^, and by electroosmotic flow encouraged by the narrow capillary geometry^46^.

The multiple sharp transients observed after the bulk oxidation peak can be assigned to oxidation of *in situ* generated nanoparticles during oxidation-reduction cycling. The observed intensity and the duration of these transients can explained based on one or more of the following: (i) Some of the metal particles may detach from the catalytic surface during reduction and form a colloidal solution^38^. As the oxidation onset potential is reached, any adsorbed particles undergo bulk oxidation, while detached particles may take time to diffuse back to the surface, making them appear later in the scan. (ii) Particles that detach from the surface during oxidation may undergo multiple surface collisions as they are incrementally oxidized^47–49^. (iii) Different shaped metal particles may be oxidized at different thresholds above the bulk oxidation potential. It has been reported elsewhere that oxidation potential is dependent on nanoparticle size^50,51^.

### Visual evidence and identity of *in situ* generated nanoparticles

**Figure 6:**
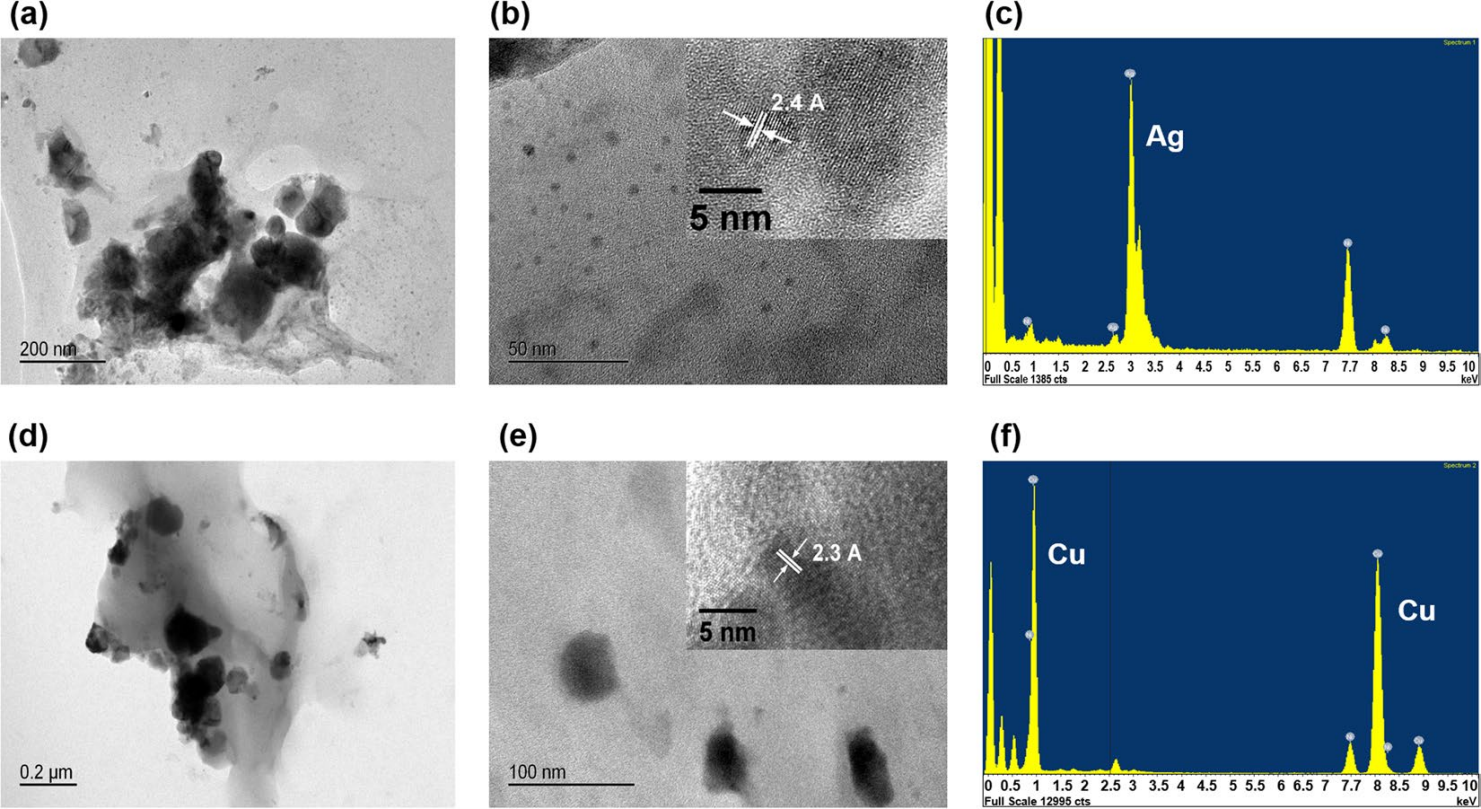
TEM images of aggregated Ag and Cu nanoparticles in the meniscus and the tip. (**a**) & (**b**) Ag NPs created at GC electrode. (**c**) EDXS analysis of particles shown in (**a**). (**d**) & (**e**) Cu NPs created at GC electrode. (**f**) EDXS analysis of particles shown in (**e**). The insets clearly indicated the interplanar spacing.

For further evidence of *in situ* generation of nanoparticles, we used TEM to image the contents of the solution and of the electrode surface. After recording periodic CVs for ~130 min, we dispensed the contents of the electrolyte at the tip (1–2 µL) onto a nickel-based TEM grid for imaging. High-resolution TEM images are shown in Fig. 6. These images show a distribution of nanoparticles of different sizes. Similar results were obtained with both Ag/AgCl QRCEs and Cu/CuCl_2_ QRCEs. Additionally, electron dispersive x-ray spectroscopy (EDXS) analysis of the particles reveals the NP composition in each solution to be silver or copper, respectively. This visual evidence is compatible with our hypothesis of NPs generated *in situ*.

### Deposition and dissolution behavior of *in situ* generated nanoparticles

**Figure 7:**
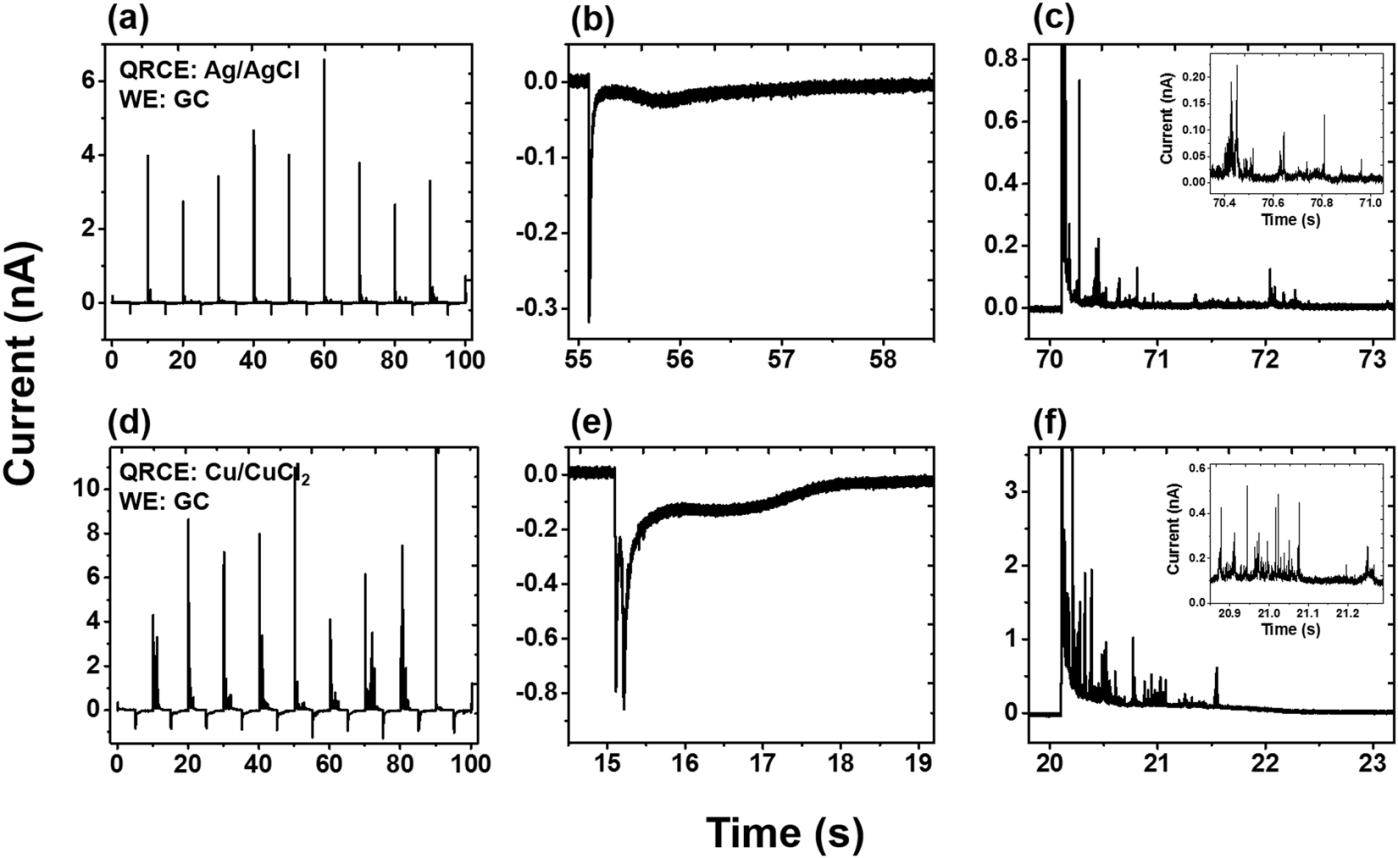
Chronoamperometric data collected on GC working electrode vs Ag/AgCl QRCE after 130 min to show distinct oxidation and reduction characteristics. (**a**) *i-t* trace recorded for 100 s (**b**) an expanded trace at −0.3 V (**c**) an expanded trace at +0.4 V. The *i-t* traces recorded vs Cu/CuCl_2_ (**d**) for 100 s (**e**) an expanded trace at −0.3 V (**f**) an expanded trace at +0.4 V.

To further investigate the oxidation and reduction behavior of *in situ* generated nanoparticles, chronoamperometric experiments were performed 80–100 min after initial contact, as shown in Fig. 7. (Initial chronoamperometric traces did not show any significant oxidation or reduction current and are shown in Fig. S3.) The potential was switched between +0.4 V and −0.3 V every 5 s, using a GC electrode with Ag/AgCl and  Cu/CuCl_2_ QRCE electrodes. When the potential is switched to −0.3 V, there is a characteristic spike due to capacitive charging. After a short delay, (~1 s), we observe a shallow peak, which corresponds to reduction of Ag^+^ into metallic silver. When the potential switches to +0.4 V, a large oxidation current is observed. Here, the transient oxidation peak is ~4 nA, which is 5 times larger than the negative switching transient (−0.8 nA), which suggests that the initial oxidation peak is due both to charging current and to bulk oxidation of Ag. The bulk oxidation of Ag occurs quickly and is too fast to separate from the capacitive transient; this oxidation occurs much faster than the earlier reduction, due to silver particles being adsorbed on the GC surface, as compared to the mass transport originally required of silver ions. After the initial peak, further oxidation spikes were observed, before the background current settles to a constant value, as shown in Fig. 7(c). These current transients suggest the oxidation may involve multiple discrete collisions with the electrode surface, as a NP is incrementally oxidized. (Similar behavior has been observed using solutions of silver nanoparticles^47^).

Figure 7(d–f) shows the *i-t* traces recorded for Cu/CuCl_2_ QRCE, which is qualitatively similar to Ag/AgCl QRCE. However, under similar conditions, we observe many more transient oxidation events for copper as compared with silver. Additionally, there is a second sharp negative current transient observed before the shallow reduction peak, which was absent with the Ag/AgCl QRCE. The higher intensity of the oxidation peaks observed for Cu particles can be attributed to higher stripping rate of the QRCE in chloride medium. We attribute the second reduction peak to the deposition of copper chloride complexes. The higher negative charge associated with the complex will be repelled from the negatively charged electrode surface and this hindered diffusion can cause a delay in the reduction peak in the *i-t* trace.

While we have presented clear evidence of nanoparticle generation, it could be reasonable to ask whether these NPs are caused by physical forces rather than electrochemical effects. To verify that the physical instability of the chlorinated QRCE surface is not the main source of contamination, we carried out a similar set of experiments with bare non-chlorinated Ag and Cu wires inserted as QRCEs. Both wires were polished using a fine grit paper to remove any oxide formed on the surface and rinsed thoroughly with DI water before use. The observed deposition and dissolution behavior (Fig. S4) is similar to Ag/AgCl and Cu/CuCl_2_, indicating that the Ag^+^ and Cu^2+^ sources are not from the mechanical removal of AgCl and CuCl_2_ layers.

### Nucleation and coulombic efficiency

To better appreciate how the unintended deposition of metal nanoparticles will evolve, it would be useful to study their nucleation mechanisms. Using the transient chronoamperometric reduction curves from a GC working electrode (Fig. 7), we sought to understand the nucleation of the Ag and Cu nanoparticles by applying the Scharifker–Hills (S–H) model. The rise and fall of the electrodeposition current is often attributed to a transition from spherical diffusion into slower planar diffusion, as the diffusion radii from multiple growing particles eventually overlap. The S–H model describes two important cases, corresponding to instantaneous and progressive nucleation^52–54^. Instantaneous nucleation refers to a scenario in which nucleation occurs quickly and a large number of growing particles occupy many active sites. The second scenario is progressive nucleation, in which the number of nucleation sites increases over time. The chronoamperometric curves are normalized by their values at the peak of the reduction spike, *i*_*m*_ and *t*_*m*_.

**Figure 8:**
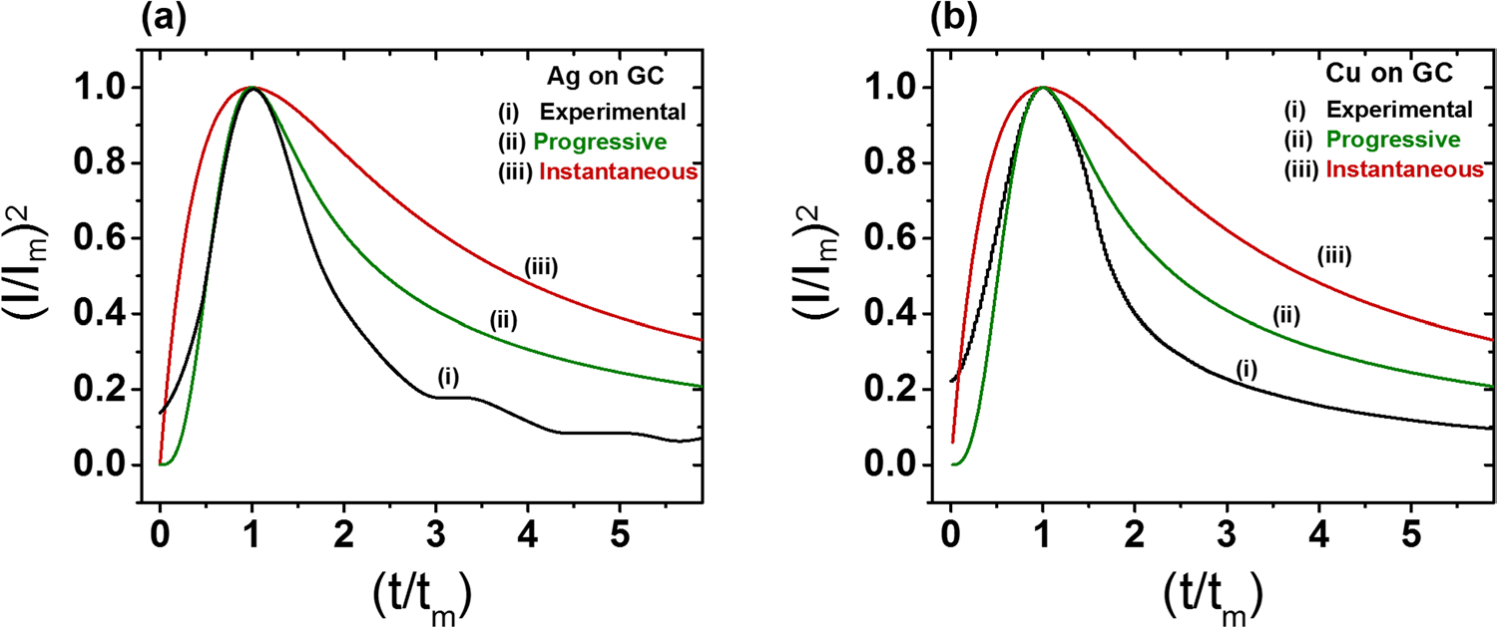
Dimensionless current vs time plots for the deposition of (**a**) Ag (**b**) Cu on GC electrode. Curve (i) shows experimental data, Curves (ii) and (iii) shows models for the limiting cases of progressive and instantaneous nuclei growth based on the Scharifker–Hills (S–H) method^52^.

The experimental and theoretical curves for Ag and Cu deposition are shown in Fig. 8. The two equations describe instantaneous and progressive models are given in the Supplementary Information. It is clear that the nucleation is progressive, as the measured current decays much faster than the instantaneous model; and in fact, it is even faster than the classical progressive model. Similar observations have been reported elsewhere, where they were attributed to competing reactions and non-uniform distributions of nucleation sites^55,56^. In our datasets, we hypothesize that the deviation is due to rate-controlling processes arise involving adatom incorporation and release at the lattice^57^, and aggregative growth^58,59^. A deeper consideration of the nucleation kinetics is reserved for future studies.

While analyzing the deposition process, we also utilized the *i-t* curves (Fig. 7) to investigate the coulombic efficiency (CE) of the particle oxidation and deposition. The total charge deposited and oxidized was calculated from the area under the curve over the period of 100 s, and the overall efficiency was found to be quite close to 100%. The CE even exceeds full efficiency for some individual cycles, indicating the presence of other nanoparticles in the vicinity of the electrode surface (Fig. S5).

### Effect of working electrodes and electrolytes

We repeated the experiment shown in Fig. 4 with polycrystalline Pt to investigate the interference from the QRCE on a different electrode surface. As usual, the CVs initially showed no redox activity and as the time progressed, characteristic stripping behavior was observed for both QRCEs (Fig. S6). One notable difference is the absence of spike/current transients after bulk oxidation. The absence of current transients suggests stronger adsorption of the particles onto the Pt surface compared to GC and complete oxidation (no partial oxidation) of particles due to faster charge transfer kinetics associated with adsorbed species on Pt. Similarly, chromoamperometric results with Pt did not show any significant current transients at oxidation potentials, nor did we observe a shallower peak at reducing potentials (Fig. S7). Instead, faster kinetics led to both oxidation and reduction currents being incorporated into larger voltage switching transient peaks. Additionally, TEM imaging of the meniscus solution did not find any particles. These results suggest the nanoparticle oxidation and reduction kinetics are very fast on a Pt working electrode, and there were no detached particles during the measurements.

Thus far, all of our experiments have used chloride electrolytes. To explore the impact of the electrolyte anion, we tested both HPO_4_^−^ (phosphate) and SO_4_^2−^ (sulfate) solutions. Both showed *in situ* generation of nanoparticles and qualitatively similar deposition-dissolution behavior to the chloride electrolyte. Chloride buffers with both acidic and basic pH also yielded similar results. Based on our experiments, the key factors of the observed interference are the small volume, close proximity of the electrodes, and the time taken for the measurements. Adjustments of the electrode distance, electrolyte volume and the measurement time can reduce these effects, but this may be in conflict with competing goals of miniaturizing a device and maximizing its sensitivity.

## Conclusions

In this study, we have described *in situ* generation of metal nanoparticles in a confined electrochemical cell as a result of redox byproducts at the QRCE which interfere with the working electrode. Using two commonly used QRCEs (Ag/AgCl and Cu/CuCl_2_), we demonstrated clear evidence of the generation of metal nanoparticles. Furthermore, we showed the origin of the metal ions is not due to physical removal of loosely plated surface layers, but rather to redox reactions at the QRCE, which suggests this behavior cannot be avoided by changing the electrode preparation. Both cyclic voltammetry and chronoamperometry provided useful information related to the deposition-dissolution behavior and nucleation of nanoparticles forming at the WE. Electron microscopy provided visual evidence of *in situ* generated particles, and confirmed their metal composition. This work highlights the importance of careful geometric design of electrochemical cells, and it re-enforces some of the challenges in designing miniaturized electrochemical systems. It is critical to recognize that metal nanoparticles can be generated even in the absence of added precursors, as these particles can distort event statistics and interfere with nanoelectrochemical experiments, particularly in increasingly popular nano-impact electrochemical methods. These conclusions similarly apply to designs of nanogap redox cycling platforms, tunneling junctions, and electrochemical lab-on-a-chip devices, where metal ions generated at the QRCE could potentially result in spurious signals or short circuits. Other emerging QRCEs, such as Pd/H_2_, could potentially avoid such complications with metal ion generation^60^ and, if their potentials prove sufficiently stable, they may be advantageous for future microscale electrochemical platforms.

## Methods

### Chemicals and materials

All solutions were prepared using deionized water (resistivity ∼18.2 MΩ cm at 25 °C) obtained from Milli-Q water purification system. Lithium chloride anhydrous (99% reagent plus) was purchased from Sigma Aldrich and used without further purification.

### Electrodes and pipette preparation

Glassy carbon (3.0 mm diameter) and platinum (1.6 mm diameter) electrodes were purchased from Bioanalytical Systems, Inc. These electrodes were polished using polishing alumina (0.05 µm) on fine grit pads (MF-1043) to obtain mirror smooth surfaces before all experiments.

Ag/AgCl QRCE electrodes were prepared by chlorinating a silver wire (99.9%, metal basis, Alfa Aesar SN#12187) in bleach for ~5 h. These Ag/AgCl electrodes were also compared with the electrodes made by an electrochemical plating method^61^. Both methods yielded identical results. Cu/CuCl_2_ reference electrodes were fabricated by electroplating the Cu wire (99.99% metal basis, Alfa Aeser SN #10972) as described elsewhere^62^. All QRCEs were thoroughly rinsed with deionized water before use.

### Fabrication of pipettes

Quartz capillaries with filaments (#QF 100-50-7.5 Sutter Instruments Co.) were pulled from a CO_2_ laser puller (Model P-2000 Sutter Instruments Co.) to obtain pipettes for the experiments according to the following two line recipe, (Line1 Heat: 700 Filament: 5 velocity: 35 delay: 150 Pull: 75. Line2 Heat: 700 Filament: 0 velocity: 15 delay: 128 Pull: 200) This recipe produced pipettes with tips with ~40 nm inner diameter.

### Electrochemical measurements

All electrochemical measurements were performed in a faraday cage (Gamry VistaShield) placed on a vibration isolated table (Newport RS 4000) to minimize electrical and mechanical noise. All experiments used a custom scanning nanopipette meniscus contact system, as illustrated in Fig. 1. A nanopositioning stage (Mad City Labs Nano-3D200) was used to bring the pipette closer to the working electrode to establish a meniscus contact. Scanning electron microscopy (SEM) images of the tips before and after the experiment are shown in Fig. S1, showing that the tips are not damaged during the experiment.

We made efforts to minimize the time elapsed between the initial contact of the QRCE with the electrolyte in the capillary and the meniscus contact in order to minimize dissolution of silver ions into the solution. In a typical experiment, this time is less than 10 min. The distance between the QRCE and the tip was 2.5 ± 0.2 cm. Similarly to SECCM experiments, it is important to to minimize evaporation, for example by using humidified cells^40,63,64^ or by adding a layer of silicone oil to the solution inside the capillary^65^. We took care to maintain high humidity in the faraday cage, and our pipette holder has a gasket which seals the non-pulled side of the capillary. No significant solution loss was observed after the experiments.

Current measurements were performed with a custom low-noise current amplifier having a gain of 50 MΩ and a signal bandwidth of approximately 3 kHz. The signal was digitized by a custom data acquisition circuit, and transferred to a computer through a USB 3.0 FPGA module (Opal Kelly). The cyclic voltammetry and pulsed protocols were implemented using custom python scripts. Data were plotted and analyzed using Matlab and OriginPro 9.1. All data except Figs 3 and 7 presented are filtered using a Savitzky–Golay filter.

### Nanoparticle imaging and characterization

*In situ* generated nanoparticles were imaged with Transmission Electron Microscopy (TEM, JEOL 2100F). Samples for TEM images were prepared by dispensing a drop of liquid from the tip (after retracting from the electrode meniscus contact) onto a carbon film supported by a nickel grid (Ted Pella, 01844N). Energy dispersive X-ray spectroscopy (EDXS) analysis was also performed during TEM analysis to determine the composition of the nanoparticles.

### Data availability

The datasets generated during and/or analyzed during the current study are available from the corresponding author on reasonable request.

## Electronic supplementary material

Supporting Information
