# Supplementary material for: Quasi-reference electrodes in confined electrochemical cells can result in in situ production of metallic nanoparticles
Source: Sci Rep. 2018 Jan 31;8:1965. doi: 10.1038/s41598-018-20412-2 (PMC5792608; doi:10.1038/s41598-018-20412-2)
Supplement: Supplementary file 1 — Supporting Information [file 41598_2018_20412_MOESM1_ESM.pdf]

Supporting information for

Quasi-reference electrodes in confined electrochemical cells can result in *in situ* production of metallic nanoparticles

Rukshan T. Perera, Jacob K. Rosenstein

School of Engineering, Brown University, 184 Hope Street. Providence. RI 02912, USA

E-mail: *jacob_rosenstein@brown.edu; rukshan@brown.edu*

Contents

1. Preparation of nanopipettes 2
2. Samples of integrated *i-t* curves used to construct charge-vs-time-elapsed plots 3
3. Initial chronoamperometric traces for Ag/AgCl and Cu/$CuCl_2$ on GC 4
4. Scharifker-Hills model 5
5. Nanoparticle generation with non-chlorinated QRCEs 6
6. Coulombic efficiency of Ag deposition 7
7. Cyclic voltammograms for Ag/AgCl and Cu/$CuCl_2$ QRCEs on Pt WE. 8
8. Chronoamperometric traces for Ag/AgCl and Cu/$CuCl_2$ QRCEs on Pt WE 9

1. Preparation of nanopipettes

Quartz capillaries with ID=0.5mm and OD=1.0mm were used to fabricate nanopipettes using a CO2-laser pipette puller (P-2000, Sutter Instrument, USA). Nanopipettes with inner diameter ~40 nm and outer diameter ~100 nm were prepared using a two line recipe:

Line1: Heat: 700 Filament: 5 velocity: 35 delay: 150 Pull: 75.

Line2: Heat: 700 Filament: 0 velocity: 15 delay: 128 Pull: 20

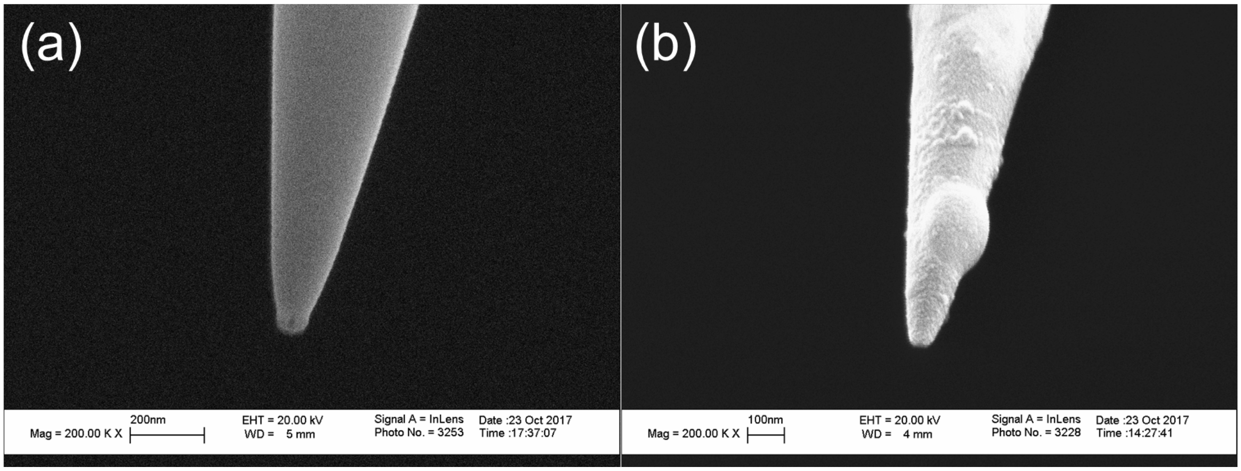

Figure S1. SEM images of quartz pipette tips (a) before and (b) after the experiment show the tip is not broken during the course of the experiment. The two pipettes are complementary and were pulled from the same capillary tube for comparison. After the experiment, some aggregated salt (LiCl) is visible after rinsing and drying the pipette in preparation for SEM imaging.

2. Samples of integrated *i-t* curves used to construct charge-vs-time-elapsed plots

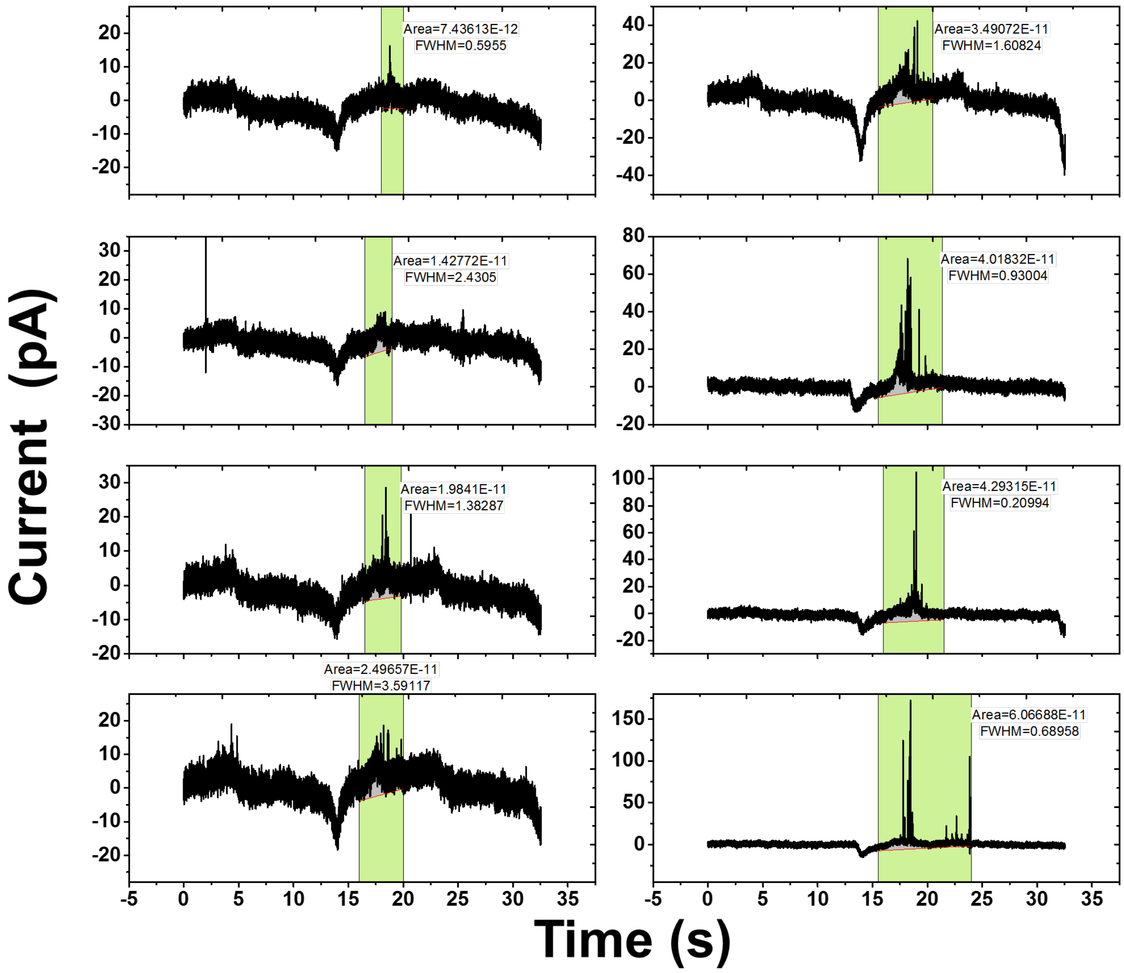

Figure S2. Representative *i-t* curves obtained from cyclic voltammograms to calculate the area under the oxidation peaks. The CVs are recorded at 0.1 V/s. The highlighted area indicates the integrated section and the grey area shows the integrated charge area.

3. **Initial chronoamperometric traces for Ag/AgCl and $Cu/CuCl_2$ on GC**

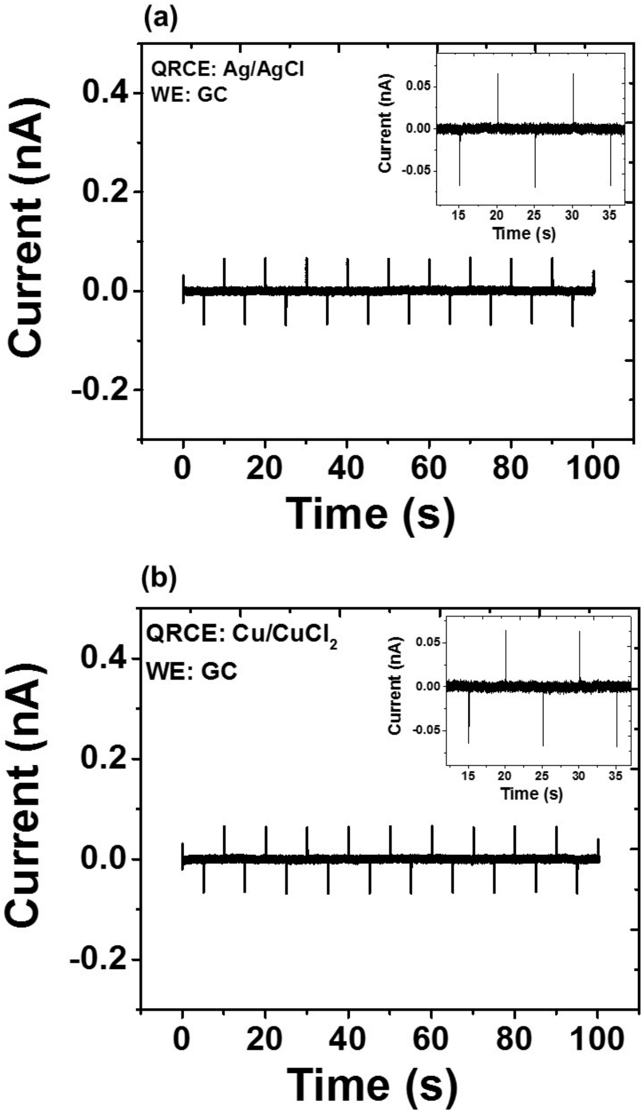

Figure S3. Initial chronoamperograms with (a) Ag/AgCl QCRE (b) $Cu/CuCl_2$ QCRE do not show significant oxidation or reduction on GC electrode. The voltage is switched between 0.4 V and -0.3 V in 5 second intervals, while the current is measured.

4. Scharifker-Hills model

The Scharifker-Hills model describes two models for particle nucleation. Equation (1) describes instantaneous nucleation, while progressive nucleation is described by equation (2). The terms, i and t represents the current and time. The model is normalized by i_m and t_m, which are the coordinates of the maximum recorded current.

$$\left(\frac{i}{i_m}\right)^2 = 1.9542\frac{t_m}{t}\left\{1 - \exp\left[-1.2564\left(\frac{t}{t_m}\right)\right]\right\}^2 \quad (1)$$

$$\left(\frac{i}{i_m}\right)^2 = 1.2254\frac{t_m}{t}\left\{1 - \exp\left[-1.2564\left(\frac{t}{t_m}\right)\right]^2\right\}^2 \quad (2)$$

5. Nanoparticle generation with non-chlorinated QRCEs

Fig. S5 presents CVs from experiments using non-chlorinated QRCEs (cleaned bare Ag or Cu wires). This supports our belief that the source of oxidized silver in the electrochemical cell is not due to mechanical instabilities of the chlorinated outer layer of the QRCE.

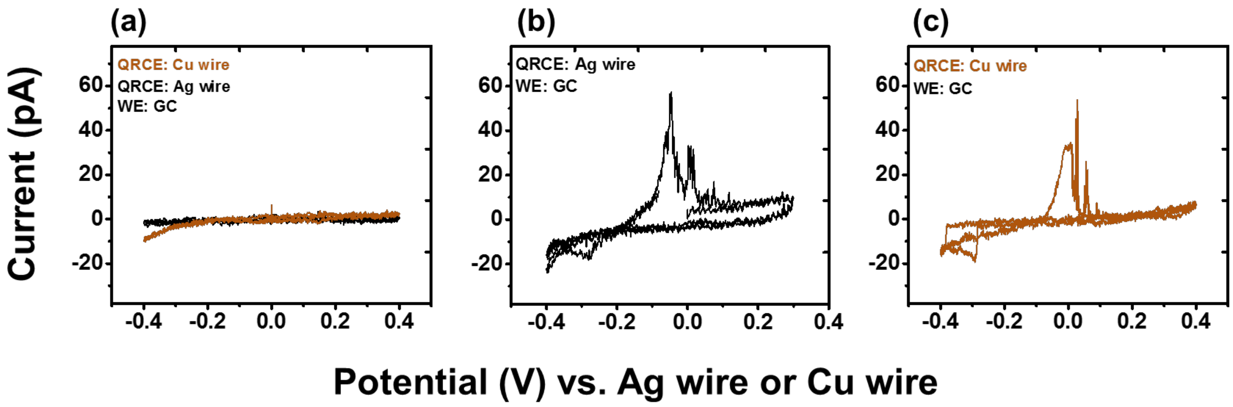

Figure S4. Comparison of cyclic voltammograms recorded on GC working electrode after ~60 mins using non-chlorinated Ag wire and Cu wire QRCEs. This suggests that the deposition and dissolution is not due to physical stripping of AgCl or $CuCl_2$ layer. CVs are recorded (a) after initial contact (b) vs Ag after ~60 min (c) vs Cu after ~60 min. All voltammograms were recorded at 0.1 V/s.

6. Coulombic efficiency of Ag deposition

Fig. 7 (a) in the main text presents chronoamperometric data collected with a GC working electrode. In this experiment, the voltage is altered between -0.3 V and +0.4 V in 5 second intervals We assume that the current corresponding to the first upward transient represents capacitive charging, and its charge is subtracted from all cycles. Coulombic efficiency was calculated as CE = oxidation charge/deposition charge. Overall, CE was near 100%, although the CE for individual cycles varies. CE values above 100% indicate the presence of other oxidizable entities (nanoparticles) in the local environment during oxidation. This observation is consistent with other evidence of unattached nanoparticles in the vicinity of the electrode.

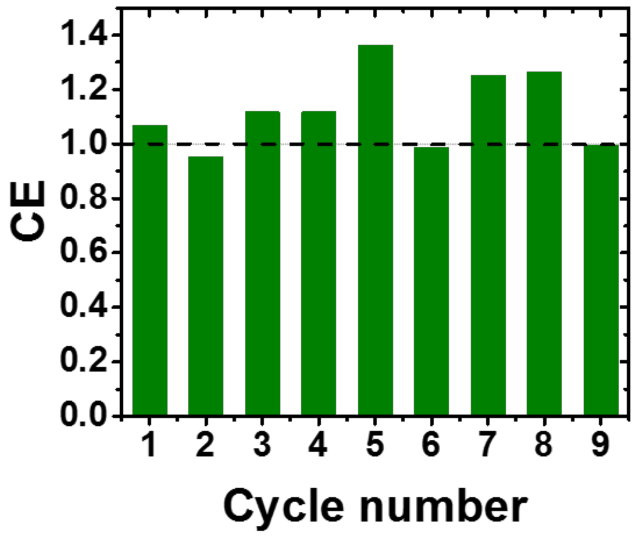

Figure S5. Coulombic efficiency for deposition and dissolution of Ag on GC electrode. vs cycle number.

7. **Cyclic voltammograms for Ag/AgCl and Cu/$CuCl_2$ QRCEs on Pt WE.**

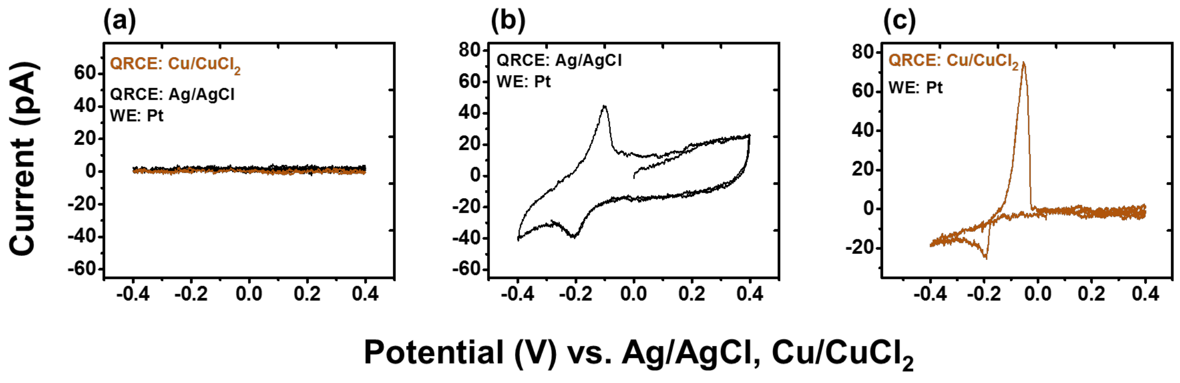

Figure S6. Comparison of cyclic voltammograms recorded on Pt working electrode after ~130 mins vs Ag/AgCl and Cu/$CuCl_2$ wire indicate the deposition and dissolution behavior. CVs are recorded (a) after initial contact for both QRCE used (b) vs Ag/AgCl after ~90 min (c) vs Cu/$CuCl_2$ after ~90 min. All voltammograms were recorded at 0.1 V/s.

8. Chronoamperometric traces for Ag/AgCl and Cu/CuCl$_2$ QRCEs on Pt WE

The *i-t* traces recorded with Pt working electrodes do not show any significant isolated NPs oxidation current transients or long shallower reduction peaks, as was seen for GC electrode. The initial peak contains the oxidation and reduction current and suggests the oxidation and reduction of silver is very fast at Pt in comparison to GC. The reduction of copper however is slower compared to silver, which is indicated by separation of Cu reduction peak from the transient current due to charging during potential switch. These results suggest that the nucleation kinetics are dependent on the catalytic surface as well as the metal being reduced (at potentials well over the over potential for reduction).

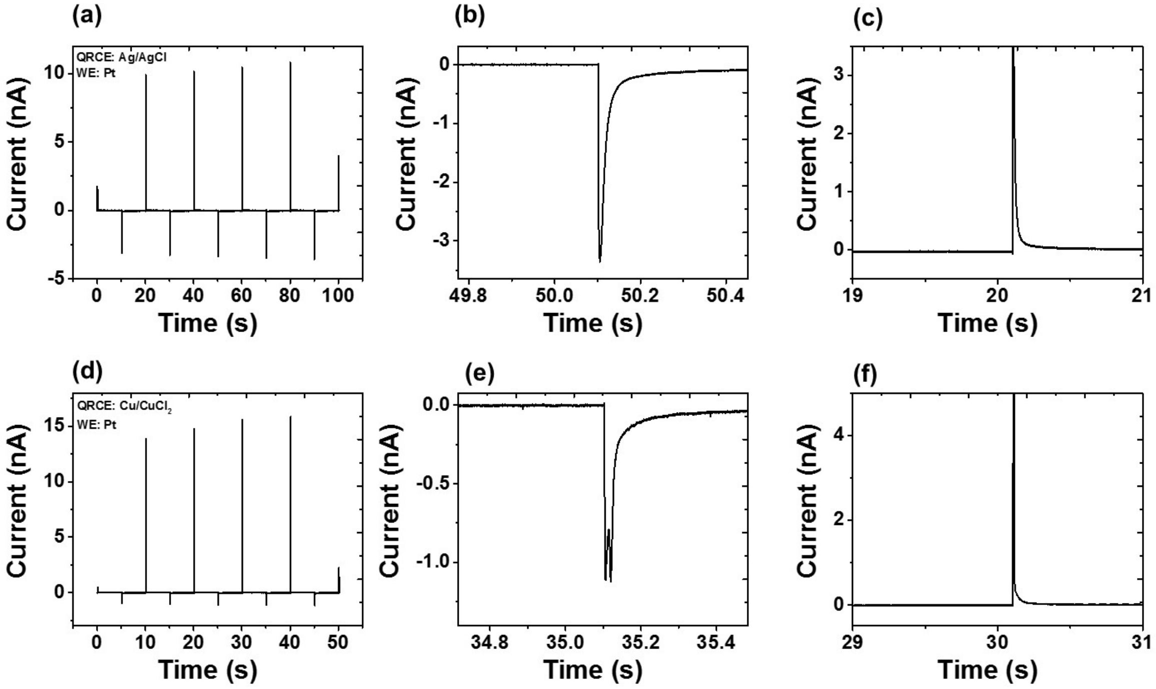

Figure S7. Chronoamperometric data collected at -0.3 V and +0.4 V with a Pt working electrode. (a) *i-t* traces recorded using Ag/AgCl QRCE, for 100 sec. (b)-(c) show expanded sections of (a). (d) Traces recorded using Cu/CuCl$_2$ QRCE. (d)-(e) show expanded sections of (d).
